# The frequency of non-motor symptoms in SCA3 and their association with disease severity and lifestyle factors

**DOI:** 10.1007/s00415-022-11441-z

**Published:** 2022-11-02

**Authors:** Holger Hengel, Peter Martus, Jennifer Faber, Paola Giunit, Hector Garcia-Moreno, Nita Solanky, Thomas Klockgether, Kathrin Reetz, Bart P. van de Warrenburg, Magda M. Santana, Patrick Silva, Inês Cunha, Luís Pereira de Almeida, Dagmar Timmann, Jon Infante, Jeroen de Vries, Manuela Lima, Paula Pires, Khalaf Bushara, Heike Jacobi, Chiadi Onyike, Jeremy D. Schmahmann, Jeannette Hübener-Schmid, Matthis Synofzik, European Spinocerebellar Ataxia Type-3/Machado-Joseph Disease Initiative (ESMI) Study Group, Ludger Schöls

**Affiliations:** 1 Department of Neurology and Hertie-Institute for Clinical Brain Research, University of Tübingen, 72076 Tübingen, Germany; 2 German Center of Neurodegenerative Diseases (DZNE), Tübingen, Germany; 3 Institute of Clinical Epidemiology and Applied Biostatistics, University of Tübingen, Tübingen, Germany; 4 German Center for Neurodegenerative Diseases (DZNE), Bonn, Germany; 5 Department of Neurology, University Hospital Bonn, Bonn, Germany; 6 Institute of Medical Genetics and Applied Genomics, University of Tübingen, Tübingen, Germany; 7 Centre for Rare Diseases, University of Tuebingen, Tübingen, Germany; 8 Ataxia Centre, Department of Clinical and Movement Neurosciences, UCL Queen Square Institute of Neurology, University College London, London, UK; 9 Department of Neurogenetics, National Hospital for Neurology and Neurosurgery, University College London Hospitals NHS Foundation Trust, London, UK; 10 Department of Neurology, RWTH Aachen University, Aachen, Germany; 11 JARA-Brain Institute Molecular Neuroscience and Neuroimaging, Forschungszentrum Jülich, Jülich, Germany; 12 Donders Institute for Brain, Cognition and Behaviour, Department of Neurology, Radboud University Medical Centre, Nijmegen, The Netherlands; 13 Center for Neuroscience and Cell Biology (CNC), University of Coimbra, Coimbra, Portugal; 14 Center for Innovation in Biomedicine and Biotechnology (CIBB), University of Coimbra, Coimbra, Portugal; 15 Faculty of Pharmacy, University of Coimbra, Coimbra, Portugal; 16 Department of Neurology and Center for Translational Neuro- and Behavioral Sciences (C-TNBS), Essen University Hospital, University of Duisburg-Essen, Essen, Germany; 17 Neurology Service, University Hospital Marqués de Valdecilla-IDIVAL, University of Cantabria (UC), Santander, Spain; 18 Centro de Investigación Biomédica en Red de Enfermedades Neurodegenerativas (CIBERNED), Madrid, Spain; 19 Department of Neurology, University Medical Centre Groningen, University of Groningen, Groningen, The Netherlands; 20 Faculdade de Ciências e Tecnologia, Universidade dos Açores, Ponta Delgada, Portugal; 21 Department of Neurology, University of Minnesota, Minneapolis, MN USA; 22 Department of Neurology, University Hospital of Heidelberg, Heidelberg, Germany; 23 Department of Psychiatry and Behavioral Sciences, Johns Hopkins University School of Medicine, Baltimore, MD USA; 24 Ataxia Center, Cognitive Behavioral Neurology Unit, Laboratory for Neuroanatomy and Cerebellar Neurobiology, Department of Neurology, Massachusetts General Hospital, Harvard Medical School, Boston, MA USA

**Keywords:** SCA3, Non-motor symptoms, Lifestyle, Physical activity

## Abstract

**Background:**

Non-motor symptoms (NMS) are a substantial burden for patients with SCA3. There are limited data on their frequency, and their relation with disease severity and activities of daily living is not clear. In addition, lifestyle may either influence or be affected by the occurrence of NMS.

**Objective:**

To characterize NMS in SCA3 and investigate possible associations with disease severity and lifestyle factors.

**Methods:**

In a prospective cohort study, we performed a cross-sectional analysis of NMS in 227 SCA3 patients, 42 pre-ataxic mutation carriers, and 112 controls and tested for associations with SARA score, activities of daily living, and the lifestyle factors alcohol consumption, smoking and physical activity.

**Results:**

Sleep disturbance, restless legs syndrome, mild cognitive impairment, depression, bladder dysfunction and pallhypesthesia were frequent among SCA3 patients, while mainly absent in pre-ataxic mutation carriers. Except for restless legs syndrome, NMS correlated significantly with disease severity and activities of daily living. Alcohol abstinence was associated with bladder dysfunction. Patients with higher physical activity showed less cognitive impairment and fewer depressive symptoms, but these differences were not significant.

**Conclusion:**

This study revealed a clear association between disease severity and NMS, likely driven by the progression of the widespread neurodegenerative process. Associations between lifestyle and NMS can probably be attributed to the influence of NMS on lifestyle.

**Supplementary Information:**

The online version contains supplementary material available at 10.1007/s00415-022-11441-z.

## Introduction

Spinocerebellar ataxia type 3 (SCA3) is the most common dominantly inherited spinocerebellar ataxia. Clinical symptoms in SCA3 are not restricted to progressive cerebellar ataxia, but extra-cerebellar motor symptoms and non-motor symptoms (NMS) contribute to disease burden [27], and NMS may even constitute the first manifestation of the disease [8]. Frequently observed NMS in SCA3 are sleep disturbance, fatigue, restless legs syndrome (RLS), neuropathy, mild cognitive decline, depression, and bladder disturbance [7, 13, 16, 19, 21, 22, 26–28, 33]. Data on the frequencies of NMS among large SCA3 cohorts are scarce, and the correlation of NMS with disease severity is only shown in parts [32]. In addition, some of these NMS are likely to influence the functional status and the lifestyle of the patients. Vice versa, different lifestyle factors might influence the severity of NMS. Detailed knowledge and awareness of NMS in SCA3 might help to enhance symptomatic treatment for these symptoms.

In this multicentric observational study, we investigated the prevalence of NMS in 227 SCA3 patients compared to 112 healthy controls as well as in 42 pre-ataxic mutation carriers. Furthermore, we assessed the association of NMS with disease severity (SARA), functional status (activities of daily living), and their association with lifestyle factors.

## Methods

### Study cohort and data collection

Based on the European Spinocerebellar ataxia type 3/Machado-Joseph disease initiative (ESMI) cohort study, a cross-sectional analysis was performed on datasets from 227 ataxic SCA3 mutation carriers, 42 pre-ataxic carriers, and 112 age- and sex-matched healthy controls from eleven European and four associated US sites. As described earlier, ataxia severity was quantified using the Scale for the Assessment and Rating of Ataxia (SARA) [8, 29]. Functional status was evaluated by the self-reported Activities of Daily Living score (ADL) of the Friedreich's Ataxia Rating Scale (FARS) [23]. NMS were collected using the PSQI questionnaire for Sleep Quality [5], the MoCA test for cognitive deficits [20], and the PHQ-9 questionnaire for depressive symptoms [15]. The presence of RLS was evaluated according to the updated International RLS Study Group consensus criteria [2]. Urinary dysfunction and pallesthesia were assessed with the Inventory of Non-Ataxia Signs (INAS) [12].

Lifestyle data were collected as previously described [9]. Specifically, physical activity was evaluated using the short form of the International Physical Activity Questionnaire (IPAQ), and data were processed according to standard recommendations [6]. Wheelchair-bound patients were excluded from further analysis regarding physical activity, as the walking domain was not applicable. Based on the IPAQ, multiples of the resting metabolic rate (MET) minutes/week were estimated, and probands were categorized into three levels of physical activity (high, moderate and low) following the IPAQ guidelines. A moderate level of physical activity on the IPAQ approximately reflects the minimum recommendation of physical activity of the WHO [4]. Alcohol consumption was assessed in a standardized interview asking about consumption on the previous workday and over the last weekend, allowing for a rough estimation of daily alcohol consumption [11]. The study was approved by the local institutional review boards of all participating centers. Written informed consent was obtained from all study participants before enrollment.

### Statistics

**Fig. 1:**
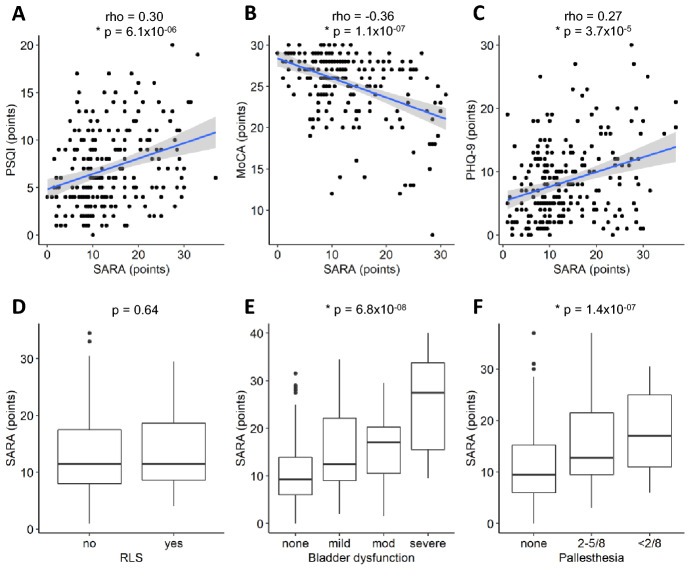
Associations between NMS and SARA scores. PSQI and PHQ-9 were positively correlated with SARA (**A**, **C**). **B** MoCA was negatively correlated with SARA. **D** RLS was independent from SARA. **E** Bladder dysfunction and (**F**) pallhypesthesia were highly significantly associated with SARA scores. *p < *0.00833 was considered significant

**Fig. 2:**
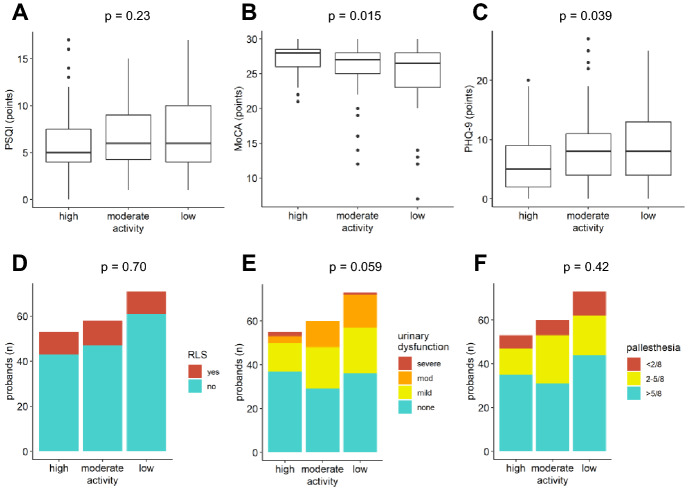
Associations between physical activity and NMS. **A** PSQI was not related to physical activity levels. **B** MoCA scores were better in subjects with higher physical activity, not significant after Bonferroni correction. **C** PHQ-9 scores were to lower in subjects with higher physical activity, not significant after Bonferroni correction. **D** Physical activity had no influence on RLS and (**E**) only minor associations with urinary dysfunction. **F** Pallhypesthesia was independent of physical activity levels. *p < *0.00833 was considered significant

**Fig. 3:**
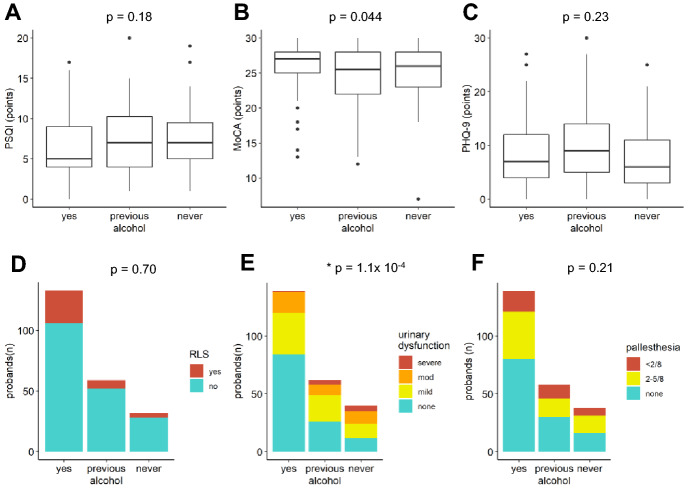
Associations between alcohol consumption and NMS. **A** PSQI, **B** MoCA, **C** PHQ-9, **D** RLS, and **F** pallhypesthesia were not associated with alcohol consumption, abstinence, or previous alcohol consumption. **E** Urinary dysfunction was significantly associated with alcohol abstinence. *p < *0.00833 was considered significant

**Fig. 4:**
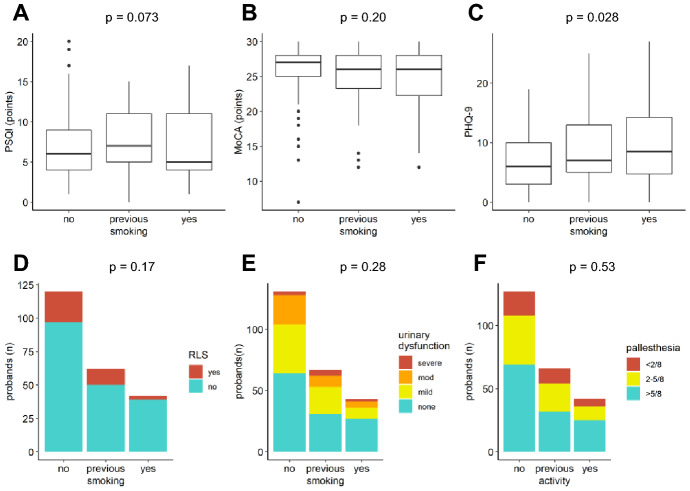
Associations between smoking and NMS. **A** PSQI, **B** MoCA, **D** RLS, **E** urinary dysfunction, **F** pallhypesthesia. PHQ-9 showed more depressive symptoms in smokers, not significant after Bonferroni correction **C**. *p < *0.00833 was considered significant

Data were analyzed using RStudio Version 1.2.5033. As none of the outcome parameters were normally distributed, the nonparametric Kruskal–Wallis test followed by the Mann–Whitney *U* test was used for group comparisons. Correlations were calculated using Spearman’s rank correlation. Bonferroni correction was applied as follows: comparison of NMS in SCA3 probands, pre-ataxic mutation carriers and controls with *m = *6 for 6 different NMS. Accordingly, *p < *0.00833 was considered significant for the prevalence of NMS shown in Table 1. Correlations with SARA, smoking, alcohol consumption und physical activity were only tested if NMS were significantly more frequent in the SCA3 group compared to the control group. Again *p < *0.00833 was used as significance level in these follow-up tests (Figs. 1, 2, 3, 4). All other analyses were considered exploratory and tested for *p < *0.00833.

**Table 1:** Characteristics of the study population and occurrence of non-motor symptoms

Demographic information	Ataxic SCA3	Pre-ataxic SCA3	Controls	*p* value pre-ataxic vs. controls	*p* value ataxic SCA3 vs. controls
Probands (*n*)	227	42	112		
Sex (f; m)	124 (51%); 119	25 (59%); 17	60 (50%); 59		
Age	51 (41.5–60.0)	34.0 (29.0–40.0)	46.5 (38.0–59.25)		
Age of onset (years)	39.0 (33.0–47.0)	NA	NA		
CAG repeat length (longer allele)	70.0 (67.0–73.0)	68 (62.0–70)	NA		
SARA	12 (8.0–19.0)	1 (0–2.0)	0 (0–0.5)		
ADL	9.0 (5.0–16.0)	0 (0–1.0)	0 (0–0)		
Alcohol (yes; previously; no)	127 (57%); 60 (27%); 35 (16%)	35 (83%); 4 (10%); 3 (7%)	96 (87%); 6 (5%); 9 (8%)		
Physical activity (high; mod; low)	51 (29%); 54 (31%); 71 (40%)	22 (56%); 8 (21%); 9 (23%)	37 (43%); 33 (39%); 15 (18%)		
Smoking (yes; previously; no)	9 (5%); 44 (24%); 130 (71%)	15 (36%); 5 (12%); 22 (52%)	9 (8%); 44 (37%); 64 (55%)		
PSQI [points]	6 (4–10)	4 (4–6.25)	4 (3–7)	0.72	*3.8 × 10^–5^
MoCA [points]	27 (24–28)	27 (26–29)	28 (27–29)	0.14	*7.2 × 10^–7^
PHQ9 [points]	7 (4–12)	5 (2–9)	3 (1–4)	0.012	*2.5 × 10^–15^
Restless legs syndrome	17%	0%	1%	1.0	*1.6 × 10^–5^
Bladder dysfunction (mild; mod; sev)	26%; 23%; 6%	3%; 3%; 0%	7%; 0%; 2%	0.65	*3.1 × 10^–13^
Pallhypesthesia (< 5/8; < 2/8)	31%; 16%	0%; 0%	6%; 2%	0.07	*1.0 × 10^–10^

* represent significant values

Data are presented as *n* (%) or median (interquartile range). *p* values were calculated using the Kruskal–Wallis test, followed by the Mann–Whitney *U* test. After Bonferroni correction for multiple testing (number of tested NMS (*m*) = 6), results were considered to be significant at *P < *0.00833. Overall comparisons using the Kruskal–Wallis tests was significant for all NMS, and followed by pairwise comparison using Mann–Whitney *U* test (*p* values listed in column 5 and 6)

*SCA3* spinocerebellar ataxia type 3; *MoCA* Montreal-Cognitive-Assessment; *PHQ9* 9-question Patient Health Questionnaire; *SARA* Scale for the Assessment and Rating of Ataxia; *ADL* Activities of Daily Living; *NA* not available; *mod* moderate; *sev* severe

## Results

Characteristics of the study population are listed in Table 1. The NMS impaired sleep quality (PSQI), cognitive deficits (MoCA), depression (PHQ-9), bladder dysfunction, restless legs syndrome (RLS) and pallhypesthesia were all highly significant more frequent in SCA3 patients compared to controls (Table 1).

In detail, poor sleep quality (PSQI > 5) was detected in 54% of SCA3 patients compared to 36% of controls and 30% in pre-ataxic mutation carriers. Mild cognitive impairment (MoCA 18–25 points) was found in 31% of SCA3 patients compared to 15% of control patients and 23% of pre-ataxic mutation carriers. Moderately reduced MoCA scores (10–17 points) were found in 5% of SCA3 and in none of the control probands or pre-ataxic mutation carriers. Two SCA3 probands and none of the control or pre-ataxic probands had severely reduced MoCA scores (< 10 points). PHQ-9 scores were higher in SCA3 probands compared to controls, indicating more depressive symptoms (median 7 points in SCA3 compared to 3 points in controls and 5 points in pre-ataxic probands). RLS was present in 17% of SCA3 probands, only 1% of healthy controls, and none of the pre-ataxic mutation carriers. Bladder dysfunction (mild, moderate, or severe) was present in 56% of SCA3 probands, while only 9% of healthy controls and 6% of pre-ataxic mutation carriers reported bladder problems. Pallhypesthesia at the ankles (< 5/8) was present in 47% of SCA3 probands, 8% of controls, and none of the pre-ataxic participants.

While none of the NMS were significantly different between pre-ataxic mutation carriers and controls, there were noticeable more depressive symptoms in pre-ataxic mutation carriers compared to controls (*p = *0.012, not significant with Bonferroni adjusted significance level 0.00833).

Based on the hypothesis that the neurodegenerative process leads to a progression of both motor and non-motor symptoms, we tested correlations of these NMS with the severity of ataxia measured by the SARA score. Correlation analysis of PSQI, MoCA and PHQ-9 with the SARA score indicated that sleep quality, cognitive deficits, and depression worsened with increasing motor disease severity (Fig. 1 A-C). Likewise, bladder dysfunction and pallhypesthesia were significantly associated with higher SARA scores (Fig. 1E, F). The presence of RLS was not associated with higher SARA scores (Fig. 1D).

Similar correlations and associations were found for the activities of daily living score (ADL). Higher (i.e., worse) ADL scores were associated with sleep disturbance, cognitive impairment, depression and pallhypesthesia (Supplementary Fig. 1). As bladder dysfunction is a part of the ADL score, no correlation analysis was calculated between bladder dysfunction and the ADL score.

Explorative analyses of the influence of age and repeat length on NMS showed a clear association between higher age and pallhypesthesia (Supplementary Fig. 2F) and an association between a shorter repeat length and pallhypesthesia. When probands were stratified for age > 45 years, there was no association between pallhypesthesia and repeat length (*p = *0.89, data not shown), suggesting that the correlation between repeat length and pallhypesthesia was due to age differences. Furthermore, higher PHQ-9 scores were correlated with longer repeats (Supplementary Fig. 3C).

Finally, we explored potential associations between non-motor symptoms and the lifestyle factors physical activity (Fig. 2), alcohol consumption (Fig. 3) and smoking (Fig. 4). We found better cognition and less depression in patients with higher activity levels. However, these differences were not statistically significant with the Bonferroni adjusted significance level of 0.00833 (Fig. 2B, C, *p* =  0.015 and *p = *0.039, respectively). Urinary dysfunction and sleep quality (PSQI) were only slightly better in patients with high activity levels (Fig. 2A, E). Pallhypesthesia and RLS were independent from physical activity levels (Fig. 2D, F).

Alcohol consumption was highly significantly associated with less bladder dysfunction (Fig. 3E,  *p* = 1.1 × 10–4). MoCA scores were slightly higher in patients consuming alcohol compared to patients who never had drunk or stopped drinking alcohol (Fig. 3B, *p* = 0.044). PSQI scores, PHQ-9 scores, pallhypesthesia and RLS were not associated with alcohol consumption (Fig. 3A, C, D, F).

Smokers had more depressive symptoms than non-smokers, but the difference was not statistically significant after Bonferroni correction (Fig. 4C, *p* = 0.028). Other NMS were not associated with smoking.

## Discussion

In this observational study, NMS including sleep disturbance, cognitive deficits, depression, RLS, bladder dysfunction and pallhypesthesia were significantly more common in SCA3 patients than in control subjects. Their frequency and severity increased in parallel with the SARA score. In pre-ataxic mutation carriers, NMS were not significantly more frequent than in healthy controls.

Autopsy studies confirmed widespread neurodegeneration in SCA3 [24, 25], in multiple CNS regions outside the cerebellum and in the peripheral nervous system, and is the likely cause of the parallel increase of NMS with disease severity assessed by the SARA score. These findings are in line with a recent study that found fatigue to increase with the severity of ataxia in SCA3 [32]. The authors suggested a bidirectional relationship between ataxia and fatigue as an explanation, but parallel worsening of both symptoms due to the parallel spread of neurodegeneration affecting multiple regions in the brain might also be a suitable explanation here. On the other hand, sensory deficits and sleep disturbances could lead to poorer performance of the SARA score. As an exception, RLS was not associated with the SARA score.

In its first description in SCA3, RLS was found to be associated with signs of peripheral neuropathy and extrapyramidal signs but not with age or repeat length [14]. This is in line with the findings from our study, where RLS did not correlate with age, the number of CAG repeats, or lifestyle factors. The strong correlation of non-motor symptoms with ADL scores could indicate that NMS lead to limitations in activities of daily living. However, the ADL score was shown to be highly correlated with the SARA score, both measuring disease progression. Thus, an association with non-motor symptoms that worsen as the disease progresses seems at hand. Interestingly, higher age was only associated with pallhypesthesia in SCA3 patients, while longer CAG repeats were associated with more depressive symptoms but better-preserved vibration sense. Peripheral neuropathy and sensory deficits have been reported earlier to be frequent in patients with shorter repeat expansions and almost not present in repeat lengths above 72 (CAG)[30]. Indeed, in our data, patients with pallhypesthesia < 2/8 all had a CAG repeat length below 73 (CAG) (Supplementary Fig. 3F). The most likely explanation is that patients with longer repeats do not reach the higher age at which neuropathy often first manifests. By stratifying for probands older than 45 years, no association between repeat length and pallhypesthesia is present; thus, strengthening the hypothesis that age but not repeat length is the critical factor here.

Regarding the observed associations between lifestyle factors and non-motor symptoms, it is not possible to establish causal relationships due to the observational nature of this study. The observed better MoCA scores and lower depression scores in patients with higher physical activity levels were not statistically significant at a Bonferroni adjusted significance level of 0.00833. This is likely due to underpowering of the study with a conservative design and conservative correction for multiple testing. Comparing only the high vs. low activity groups in an exploratory approach results in *p = *0.0047 for MoCA and *p = *0.019 for PHQ9. These differences may reflect, in part, a protective effect of physical activity for cognitive decline and depression. A risk reduction of cognitive decline, dementia and/or Alzheimer’s disease due to physical activity has been suggested multiple times [1, 17, 18]. Similarly, a protective or even therapeutic effect of physical activity on depression is well established [10, 31]. However, it is also possible that less depression and better cognition lead to more physical activity due to better drive and motivation.

Associations of alcohol consumption and NMS was limited to urinary dysfunction. A potential explanation might be the diuretic effect of alcohol. This may cause increased discomfort from diuresis due to the inhibition of vasopressin production and may lead SCA3 patients with bladder control problems to refrain from alcohol consumption.

Smoking was not significantly associated with any of the NMS. For the general population, an association between smoking and depression is well known [3]. In our study, PHQ-9 scores of SCA3 patients were indeed higher in smokers, yet the differences did not reach significance after Bonferroni correction.

In summary, our study demonstrates NMS to be frequent in SCA3 and to increase with disease severity. As most of them can be ameliorated by symptomatic treatment, awareness and explicit interrogation is important and may help to improve the care of patients with SCA3.

### Supplementary Information

Below is the link to the electronic supplementary material.

Supplementary file1 (PPTX 336 kb)
