# Correction to: The frequency of non‑motor symptoms in SCA3 and their association with disease severity and lifestyle factors

**DOI:** 10.1007/s00415-023-12064-8

**Published:** 2023-11-18

**Authors:** Holger Hengel, Peter Martus, Jennifer Faber, Paola Giunti, Hector Garcia-Moreno, Nita Solanky, Thomas Klockgether, Kathrin Reetz, Bart P. van de Warrenburg, Magda M. Santana, Patrick Silva, Inês Cunha, Luís Pereira de Almeida, Dagmar Timmann, Jon Infante, Jeroen de Vries, Manuela Lima, Paula Pires, Khalaf Bushara, Heike Jacobi, Chiadi Onyike, Jeremy D. Schmahmann, Jeannette Hübener-Schmid, Matthis Synofzik, European Spinocerebellar Ataxia Type-3/Machado-Joseph Disease Initiative (ESMI) Study Group, Ludger Schöls

**Affiliations:** 1 Department of Neurology and Hertie-Institute for Clinical Brain Research, University of Tübingen, 72076 Tübingen, Germany; 2 German Center of Neurodegenerative Diseases (DZNE), Tübingen, Germany; 3 Institute of Clinical Epidemiology and Applied Biostatistics, University of Tübingen, Tübingen, Germany; 4 German Center for Neurodegenerative Diseases (DZNE), Bonn, Germany; 5 Department of Neurology, University Hospital Bonn, Bonn, Germany; 6 Institute of Medical Genetics and Applied Genomics, University of Tübingen, Tübingen, Germany; 7 Centre for Rare Diseases, University of Tuebingen, Tübingen, Germany; 8 Ataxia Centre, Department of Clinical and Movement Neurosciences, UCL Queen Square Institute of Neurology, University College London, London, UK; 9 Department of Neurogenetics, National Hospital for Neurology and Neurosurgery, University College London Hospitals NHS Foundation Trust, London, UK; 10 Department of Neurology, RWTH Aachen University, Aachen, Germany; 11 JARA-Brain Institute Molecular Neuroscience and Neuroimaging, Forschungszentrum Jülich, Jülich, Germany; 12 Donders Institute for Brain, Cognition and Behaviour, Department of Neurology, Radboud University Medical Centre, Nijmegen, The Netherlands; 13 Center for Neuroscience and Cell Biology (CNC), University of Coimbra, Coimbra, Portugal; 14 Center for Innovation in Biomedicine and Biotechnology (CIBB), University of Coimbra, Coimbra, Portugal; 15 Faculty of Pharmacy, University of Coimbra, Coimbra, Portugal; 16 Department of Neurology and Center for Translational Neuro- and Behavioral Sciences (C-TNBS), Essen University Hospital, University of Duisburg-Essen, Essen, Germany; 17 Neurology Service, University Hospital Marqués de Valdecilla-IDIVAL, University of Cantabria (UC), Santander, Spain; 18 Centro de Investigación Biomédica en Red de Enfermedades Neurodegenerativas (CIBERNED), Madrid, Spain; 19 Department of Neurology, University Medical Centre Groningen, University of Groningen, Groningen, The Netherlands; 20 Faculdade de Ciências e Tecnologia, Universidade dos Açores, Ponta Delgada, Portugal; 21 Department of Neurology, University of Minnesota, Minneapolis, MN USA; 22 Department of Neurology, University Hospital of Heidelberg, Heidelberg, Germany; 23 Department of Psychiatry and Behavioral Sciences, Johns Hopkins University School of Medicine, Baltimore, MD USA; 24 Ataxia Center, Cognitive Behavioral Neurology Unit, Laboratory for Neuroanatomy and Cerebellar Neurobiology, Department of Neurology, Massachusetts General Hospital, Harvard Medical School, Boston, MA USA

**Correction to: Journal of Neurology (2023) 270:944–952** 10.1007/s00415-022-11441-z

In the original version of this article, the author’s name Paola Giunti was incorrectly written as Paola Giunit.

