# Supplementary material for: The frequency of non-motor symptoms in SCA3 and their association with disease severity and lifestyle factors
Source: J Neurol. 2022 Nov 2;270(2):944–52. doi: 10.1007/s00415-022-11441-z (PMC9886646; doi:10.1007/s00415-022-11441-z)
Supplement: Supplementary file 1 — Supplementary file1 (PPTX 336 kb) [file 415_2022_11441_MOESM1_ESM.pptx]

## Slide 1
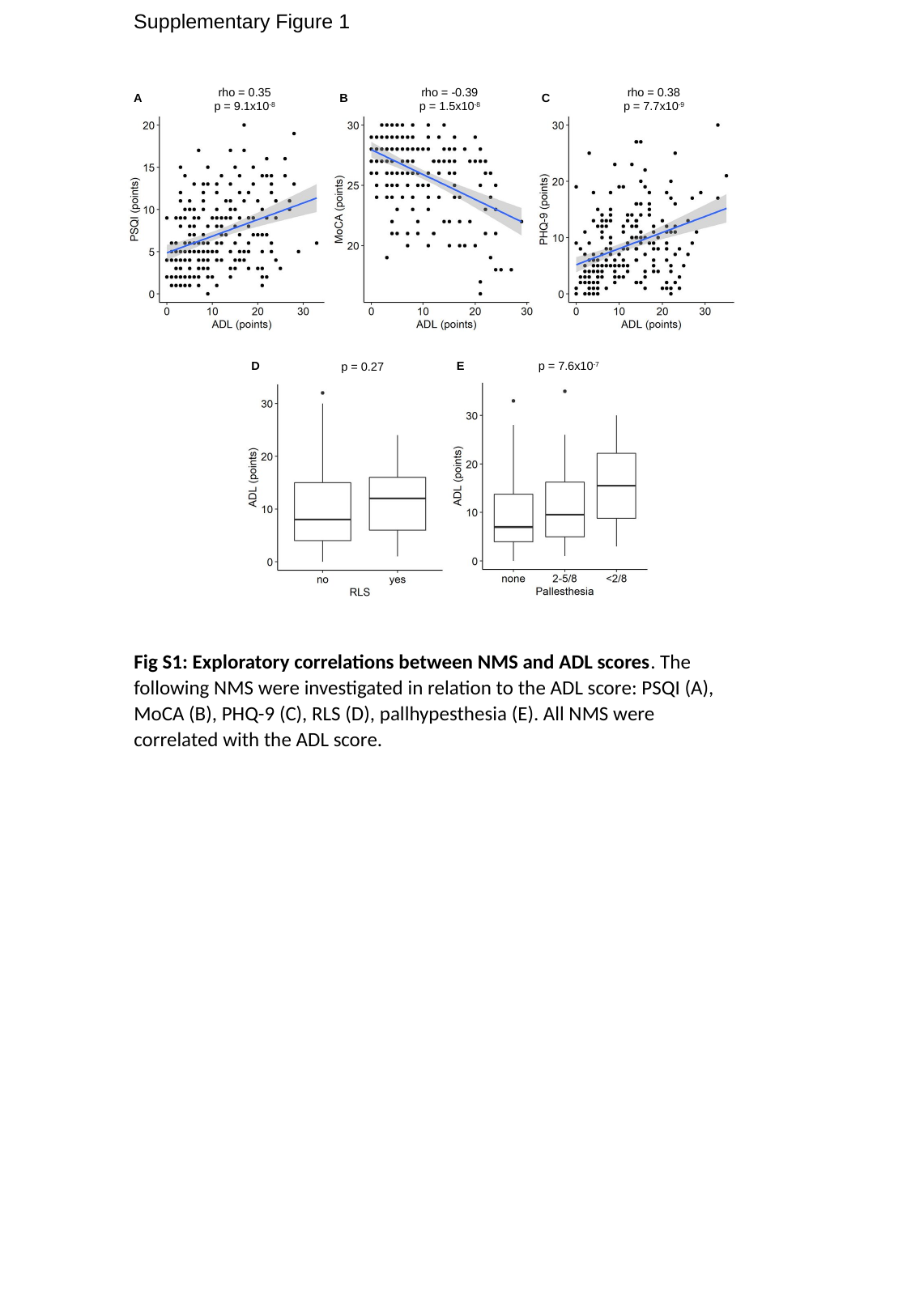

Supplementary Figure 1
rho = 0.35
p = 9.1x10^-8^
rho = -0.39
p = 1.5x10^-8^
rho = 0.38
p = 7.7x10^-9^
A
B
C
D
E
p = 7.6x10^-7^
p = 0.27
Fig S1: Exploratory correlations between NMS and ADL scores. The following NMS were investigated in relation to the ADL score: PSQI (A), MoCA (B), PHQ-9 (C), RLS (D), pallhypesthesia (E). All NMS were correlated with the ADL score.

## Slide 2
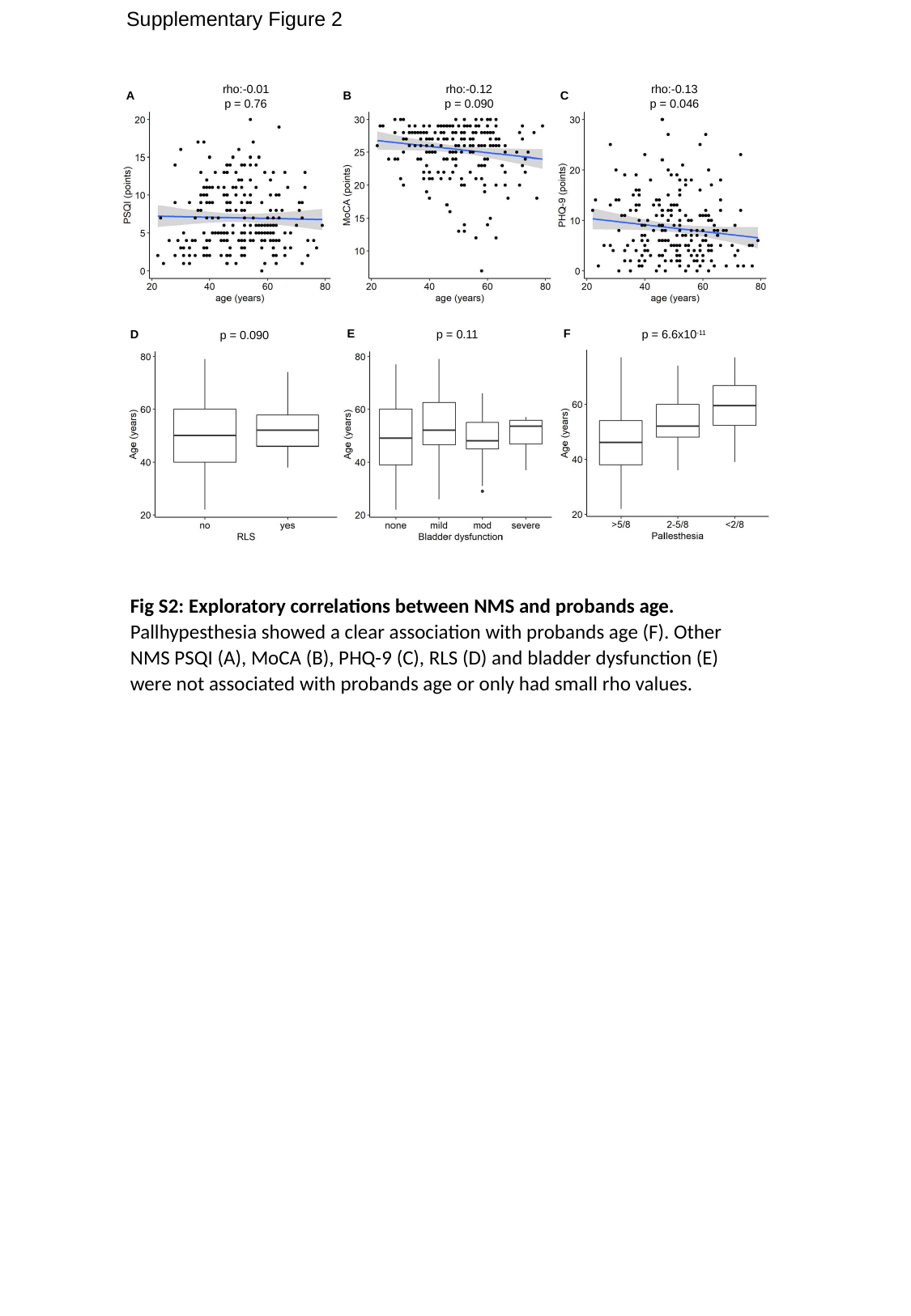

Supplementary Figure 2
rho:-0.01
p = 0.76
rho:-0.12
p = 0.090
rho:-0.13
p = 0.046
A
B
C
E
F
D
p = 0.11
p = 6.6x10^-11^
p = 0.090
Fig S2: Exploratory correlations between NMS and probands age. Pallhypesthesia showed a clear association with probands age (F). Other NMS PSQI (A), MoCA (B), PHQ-9 (C), RLS (D) and bladder dysfunction (E) were not associated with probands age or only had small rho values.

## Slide 3
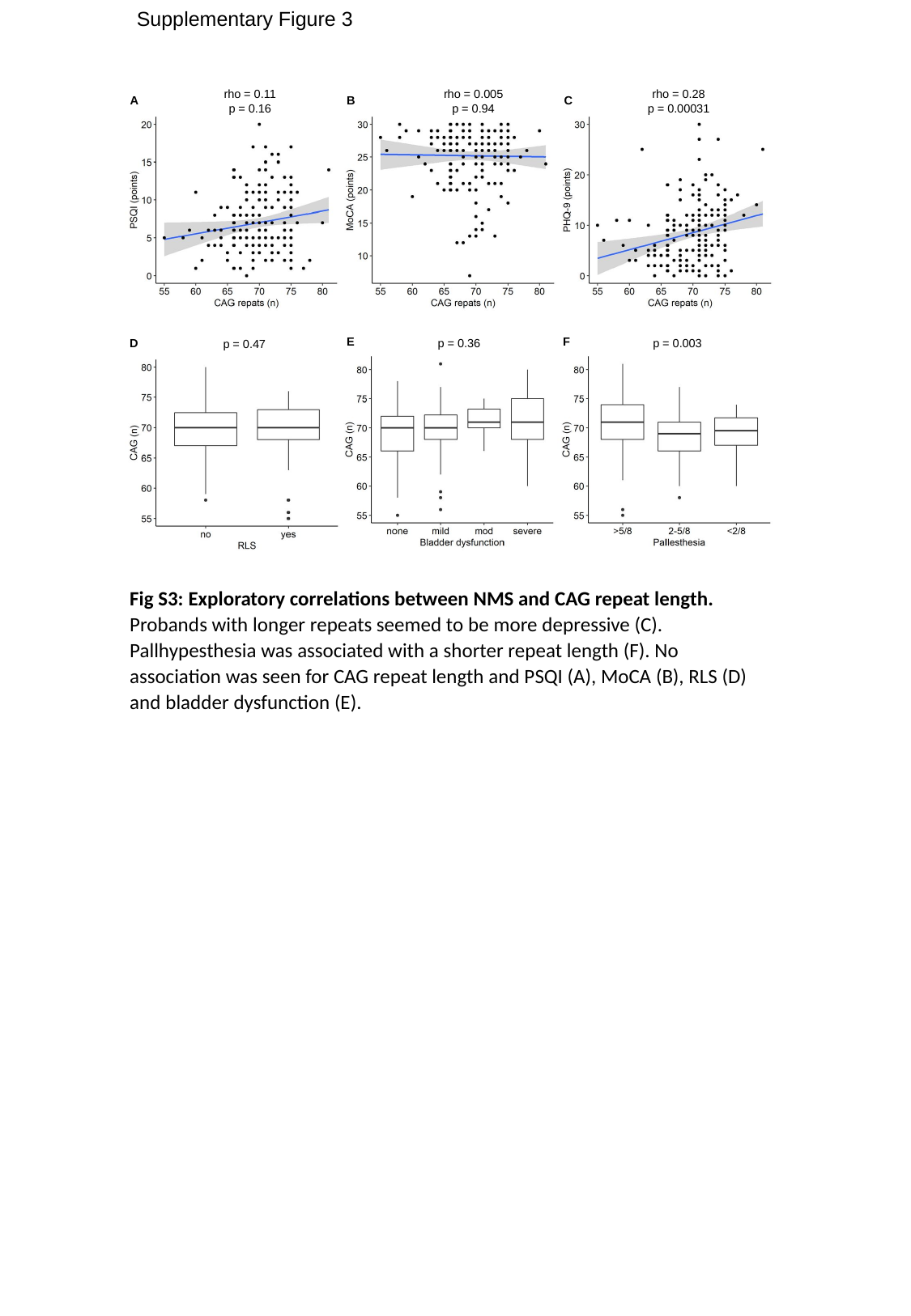

Supplementary Figure 3
rho = 0.11
p = 0.16
rho = 0.005
p = 0.94
rho = 0.28
p = 0.00031
A
B
C
E
F
D
p = 0.36
p = 0.003
p = 0.47
Fig S3: Exploratory correlations between NMS and CAG repeat length. Probands with longer repeats seemed to be more depressive (C). Pallhypesthesia was associated with a shorter repeat length (F). No association was seen for CAG repeat length and PSQI (A), MoCA (B), RLS (D) and bladder dysfunction (E).
